# A long-term investigation of the anti-hepatocarcinogenic potential of an indigenous medicine comprised *of Nigella sativa, Hemidesmus indicus and Smilax glabra*

**DOI:** 10.1186/1477-3163-5-11

**Published:** 2006-05-09

**Authors:** SS Iddamaldeniya, MI Thabrew, SMDN Wickramasinghe, N Ratnatunge, MG Thammitiyagodage

**Affiliations:** 1 Department of Biochemistry, Faculty of Medicine, University of Sri Jayawardenepura, Gangodawila, Nugegoda, Sri Lanka; 2 Department of Biochemistry and Clinical Chemistry, Faculty of Medicine, University of Kelaniya, 6, Thalagolla Road, Ragama, Sri Lanka; 3 Department of Pathology, Faculty of Medicine, University of Peradeniya, Peradeniya, Sri Lanka; 4 Animal Centre, Medical Research Institute, Colombo 08, Sri Lanka

## Abstract

**Background:**

A decoction comprised of *Nigella sativa *seeds, *Hemidesmus indicus *root bark and *Smilax glabra *rhizome is being recommended for cancer patients by a family of traditional medical practitioners of Sri Lanka. Previous investigations have demonstrated that a short term (10 weeks) treatment with the decoction can significantly inhibit diethylnitrosamine (DEN) mediated expression of Glutathione S-transferase P form (GST-P) in rat liver. The objective of the present investigation was to determine whether long term (16 months) treatment with the decoction would be successful in inhibiting in rat livers, not only DEN- mediated expression of GST-P, but also the carcinogen mediated development of overt tumours (OT) or histopathological changes leading to tumour development (HT).

**Methods:**

Thirty-six male Wistar rats were divided into 3 groups of 12 each. Groups 1 and 2 were injected intraperitoneally (i.p) with DEN (200 mg/kg) while group 3 was injected normal saline (NS). Twenty-four hours later, decoction (DC; 6 g/kg body weight/day) was orally administered to group 1 rats, while groups 2 and 3 (DEN-control and normal control) were given distilled water (DW). Treatment with DC or DW continued for 16 months. At the end of the 9^th ^month and 16^th ^months (study 1 and study 2 respectively), six rats from each group were sacrificed, and livers observed for OT or HT, both visually and by subjecting liver sections to staining with Haemotoxylin and Eosin (H & E), Sweet's Silver stain (for reticulin fibers), Periodic Acid Schiff (PAS) staining (for glycogen), and immunohistochemical staining (for GST-P).

**Results:**

At the end of 9 months (study 1) a hepatocellular adenoma (HA) developed in one of the rats in the DEN + DW treated group (group 2). At the end of 16 months (study 2), livers of all rats of group 2 developed OT and HT. Large areas of GST-P positive foci were also observed. No OT, HT or GST-P positive foci were detected in any of the other groups.

**Conclusion:**

Protection against DEN-mediated carcinogenic changes in rat liver can be achieved by long term treatment with the DC comprised of *N. sativa *seeds, *S. glabra *rhizome and *H. indicus *root bark.

## Background

Traditional and Ayurvedic Physicians in Sri Lanka use several plant-based remedies for treatment of cancer with varying degrees of success. However, until recently, none of these preparations have been subjected to any form of scientifically controlled investigation to determine their efficacy as curative or palliate agents against cancer. A herbal remedy prescribed to cancer patients by the Jayathilake family of Ayurvedic physicians (personal communication, Ayurvedic Dr. Nimal Jayathilake) is a decoction (DC) prepared from *Nigella sativa *seeds, *Hemidesmus indicus *root and *Smilax glabra *rhizome. A recent investigation by Iddamaldeniya *et al *[1] demonstrated that short-term (10 weeks) treatment of rats with the DC can protect their livers against DEN- mediated expression of the P-isoform of Glutathione S-transferase (GST-P). The objective of the present study was to determine the long-term anti-hepatocarcinogenic effects of the DC. This was achieved by assessing the ability of the DC to inhibit diethylnitrosamine (DEN)-mediated expression of GST-P, as well as the development of overt tumours (OT) and histopathological changes leading to tumour development (HT), in livers of rats treated with the carcinogen.

## Methods

### Experimental animals

In all experiments Wistar rats (8 week old littermates, 190 ± 10 g) purchased from the Medical Research Institute, Sri Lanka (MRI), were used and maintained in a temperature controlled room (25°C ± 12°C) under 12 hours light/dark cycle (dark phase 6 p.m. to 6 a.m.). The Wistar strain that originated from the Wistar Institute of Biology, USA and introduced into CLEA, Japan were introduced to the Animal House, MRI, Sri Lanka in 1990.

Rats were fed with a standard laboratory diet containing 19% crude proteins, 3.8% fibre and 4400 kcal of energy, prepared by the Medical Research Institute, Sri Lanka, based on a formula recommended by the WHO, and water *ad libitum *[2]. The feed was prepared from ingredients purchased from several companies. Thus, Maize meal, Milk powder, Wheat flour and Mollasses were purchased from Ceylon Grain Elevators, Ceylon Milk Foods, Ceylon Wholesale Establishment, and Sugar factory, Hingurana. Sri Lanka, respectively. Fish Meal (Danish 999) was imported by the MRl, directly from Denmark. All other ingredients were supplied by S. V.K. and Sons, Colombo, Sri Lanka.

### Plant material

Dried rhizome of *Smilax glabra*, dried seed of *Nigella sativa *and dried root of *Hemidesmus indicus *were purchased locally from Nandana Ayurveda Drug Suppliers, Maharagama, Sri Lanka and identities were confirmed by the Botanist (Mr.Gunarathne Silva), Bandaranayake Memorial Ayurveda Research Institute, Navinna, Maharagama, Sri Lanka. A single batch of dried plant material purchased initially was used throughout the experimental period, to avoid batch- to-batch variations.

### Chemicals

Diethylnitrosamine (DEN) and Diaminobenzidine (DAB) were purchased from Sigma Diagnostics Inc. USA Normal Swine serum, Rabbit polyclonal anti GST -P antibody, Biotin labeled anti Rabbit IgG and Avidin Biotin-peroxidase Complex (ABC) were purchased from DAKO, Denmark. All other chemicals were purchased from Sigma Chemicals, USA.

### Preparation of the decoction

The plant decoction was prepared according to the method recommended traditionally for administration to cancer patients (information provided by Dr. N. Jayathitlake, Bandaranayake Memoria1 Ayurvedic Research lnstitute, Navinna, Maharagama, Sri Lanka.)

Twenty grams each of *Nigella sativa *(seeds), *Hemidesmus indicus *(root) and *Smilax glabra *(rhizome) were mixed and boiled in 1.6 l of distilled water and the final volume was reduced to 200 m1 by boiling over 3 hours.

### Dosage and administration of decoction

The decoction was administered at a dose of 6 g/kg/day to rats using a Sondi needle by gastric gavage method. This dose was used in all experiments, as it proved to be the most effective dose in the short term study conducted previously [1].

### Ethical approval

Ethical approval for the study was granted by the Ethical Committee of the University of Sri Jayawardenapura, Sri Lanka.

### Experimental procedure

Thirty-six Wistar rats were randomly divided into 3 groups of 12 each. Groups 1 and 2 were injected with DEN (200 mg/kg) to initiate hepatocarcinogenesis [3] while group 3 was given normal saline (NS) i.p. Twenty-four hours later the DC (6 g/kg body weight/day) was orally administered to group 1 rats, while groups 2 and 3 were given same volume (3 ml) of distilled water (DW). Oral feeding continued for two weeks after which all rats were subjected to 2/3 partial hepatectomy (PH) by the technique recommended by Higgins and Anderson [4], to promote hepatocarcinogenesis. On the following day, oral feeding was resumed and continued for a total of 16 months. At end of the 9^th ^months (study 1) and 16^th ^month (study 2), six rats from each group respectively, were sacrificed and livers examined for OT and tumour like lesions (TL). Samples of livers were also excised and processed for assessment of DEN-mediated histopathological changes related to hepatocarcinogenesis (HT) as well as GST-P expression.

### Tissue processing and staining

At autopsy, livers were excised from all animals, and slices of 2–3 mm thick (six slices of liver, two each from the right posterior, right anterior and caudate lobes) were cut with a surgical blade, fixed in 10% phosphate buffered formalin. The samples were procssed using a tissue processor and embedded in paraffin. Paraffin blocks were cut using a micro tome cutter. Three sets of the liver sections from study 1 were subjected to Haemotoxylin and Eosin (H & E) staining, Silver staining for reticulin fibers, and Immunohistochemical staining for Glutathione S-transferase P form (GST -P). Sections from rats in study 2 were stained with all the above stains, as well as the Periodic Acid Schiff (PAS) stain (with and without Diastase) for detection of glycogen.

The method described by Alan and Ian [5] was employed in making H & E stained sections. The Silver staining method described by Gorden and Sweet [6] was employed to stain reticulin fibres in liver sections, while PAS staining was conducted according to the method described by McManus [7].

### GST -P immunohistochemistry

The Avidin Biotin peroxidase Complex (ABC) method described by Hsu *et al *[8] was used to demonstrate GST -P liver foci. Deparaffinized sections were treated with normal swine serum (1: 10), rabbit po1yc1onal anti GST -P antibody (1:150), biotin labeled anti rabbit IgG (1:300) and ABC. The sites of peroxidase binding were visualized using Diaminobenzidine (DAB). Sections were counter stained with Carazzis Haematoxylin for microscopic examination. As positive control for the specificity of anti- GST -P antibody binding human thyroid sections were used. The number of foci and number of cells/cm^2 ^were measured using an OLYMPUS research microscope (×400). The final numbers for each parameter were computed after microscopic examination of the whole area of each section.

The above parameters were quantified in the prepared liver sections by Prof. N. Ratnatunge (pathologist Faculty of Medicine, University of Peradeniya, Sri Lanka), who was blind to the identities of the samples.

### Statistical analysis

The results are expressed as Mean ± Standard Error of Mean (S.E.M). The significance of difference in the number of GST-P positive foci between the control and test groups were analyzed by the Student's t-test.

## Results

**Figure 1:**
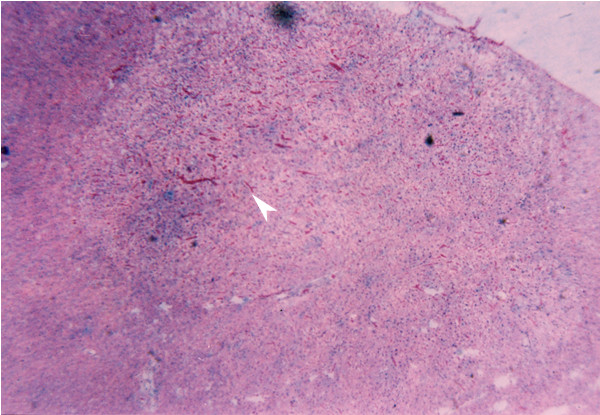
Hepatocellular Adenoma 9 months after DEN + DW treatment. H & E staining (×4)

**Figure 2:**
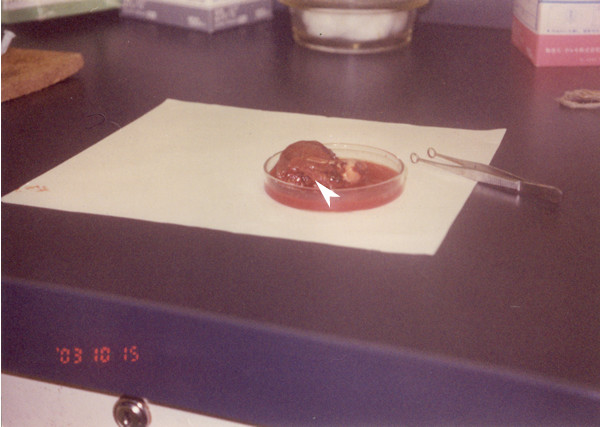
Hepatocellular carcinoma 16 months after DEN + DW treatment.

**Figure 3:**
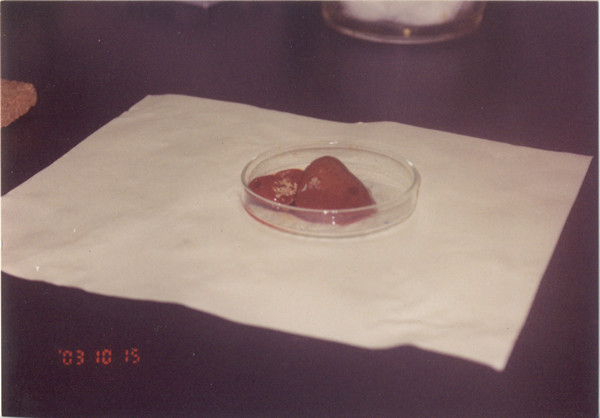
A liver from the group which received DEN + DC for 16 months.

On examination of rat livers at the end of 9 months, a hepatocellular adenoma was observed in only one of the rats in group 2 (DEN + DW) (Fig 1). However, by the end of the 16^th ^month, overt tumours (OT) or tumour like lesions (TL) were observed visually (Fig. 2) in livers of all rats in this group. In contrast, no OT or TL were observed in the post-mortem, in either rats from this group 1 (DEN+ DC) or group 3 (NS + DW), even at the end of 16 months (Fig. 3), although the livers of group 1 animals appeared to be hyper plastic, with some adhesions.

**Figure 4:**
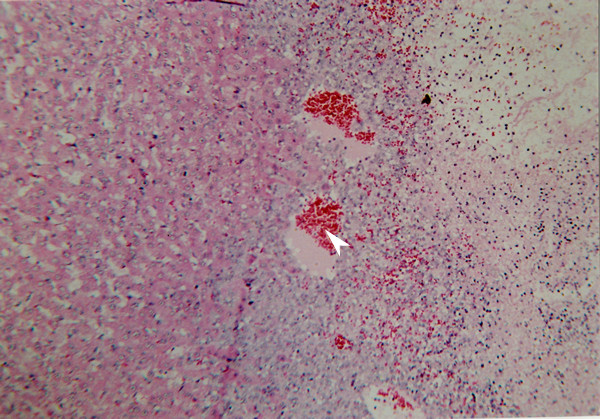
Liver section from group 2 (DEN + DW), H & E, (×10).

**Figure 5:**
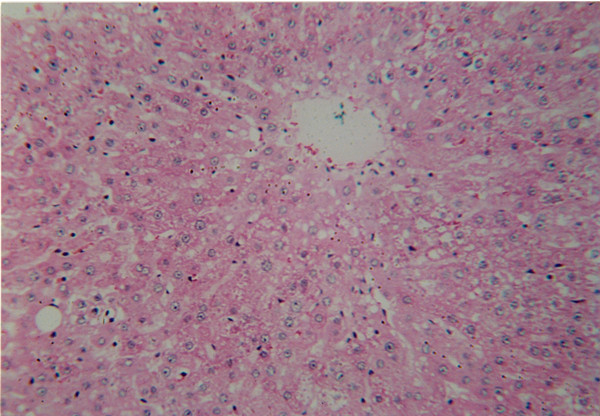
Liver section from group 1 (DEN + DC), H & E, (×10).

H & E staining revealed in all rats of group 3 (NS + DW), the typical architecture expected of normal livers, even at the end of 16 months. On the other hand, H & E staining of livers of group 2 rats (DEN + DW) at the end of 9 months, revealed severe and diffused granular degeneration and cell swelling; At the end of 16 months, growing hepatocellular carcinomas were evident in livers of all rats in this group (Fig.4). One of the most notable features observed in livers of this group, was the extensive angiogenesis. Although a mild to moderate degree of granular and vacuolar degeneration were observed in livers of group 1 (DEN + DC) rats at the end of 16 months, the degree of such degenerative changes, as well as angiogenesis in livers of this group, were of much less intensity than those seen in group 2 rats (Fig. 5).

**Figure 6:**
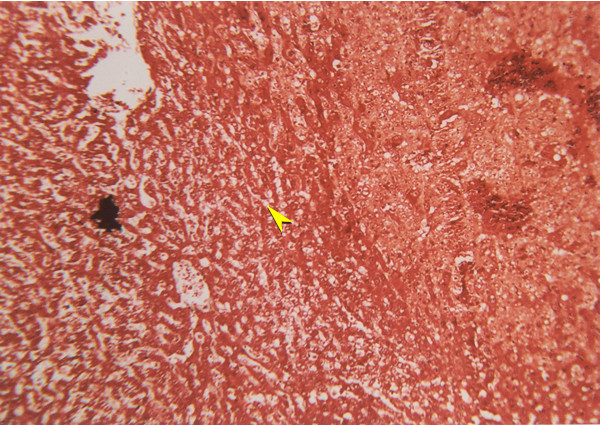
Sweet's Silver staining of group 2 liver section (×40).

**Figure 7:**
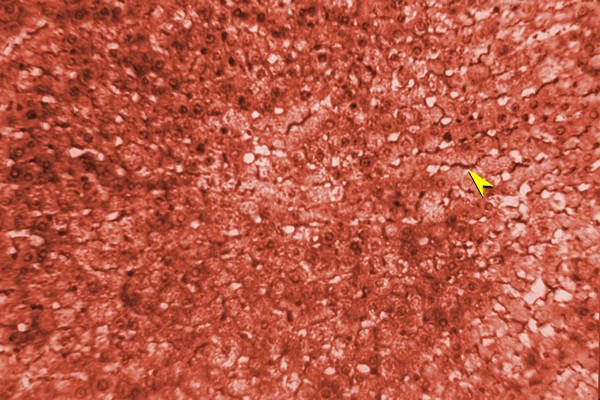
Sweet's Silver staining of group 1 liver section (×40).

As expected, silver staining revealed a normal black stained reticulin framework in livers of group 3 rats (NS + DW) throughout the experimental period. However, in group 2 (DEN + DW) animals, silver staining revealed disruption of the reticulin framework, as evident from the reduction in staining intensity in some areas. At the end of 16 months, a severe reduction of the reticulin framework was observed in rats of this group, as demonstrated by the virtual absence of silver stained reticulin fibres in the liver sections (Fig. 6). In contrast, in group 1 (DEN + DC) animals, at the end of 16 months of DC treatment, a return of the reticular framework towards normal was observed (Fig. 7).

**Figure 8:**
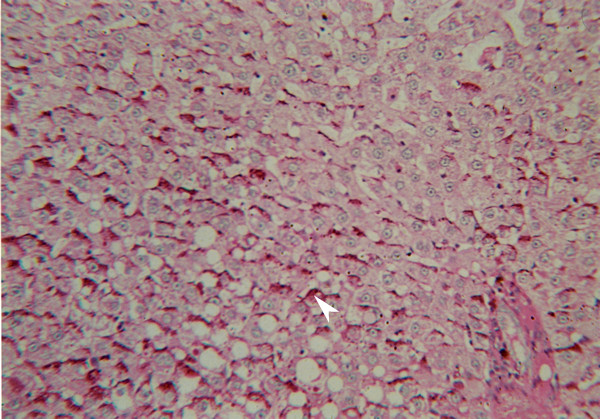
PAS staining with diastase, group 2 (×40).

Further support for the development of hepatocellular carcinomas in group 2 (DEN + DW) rats, was obtained by PAS staining. Thus, in these animals, positive staining of similar intensity was observed in liver sections exposed to this stain, both with and without diastase (Fig. 8). However, in livers of group 1 rats (DEN + DC), PAS staining without diastase was positive while PAS staining with diastase was negative.

**Figure 9:**
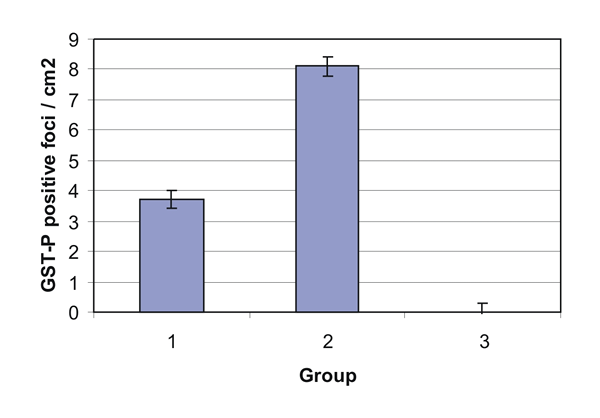
Effects of a decoction treatment for 16 months on number of DEN-mediated GST-P positive foci in Wistar rats. Group 1: DEN + Decoction. Group 2: DEN + Distilled water. Group 3: Normal saline + Distilled water

As demonstrated in the previous short- term investigation by Iddamaldeniya *et al *[1], immunohistochemical staining revealed large areas of GST-P positive foci in livers of groups 2 rats (DEN +DW), but not in rats of group 3 (NS + DW). In group 1 rats (DEN + DC), in comparison to livers of group 2 (DEN + DW) animals, there was a significant reduction (P < 0.05) in the number of GST-P positive foci (Fig 9).

## Discussion

An increasing number of investigations are being conducted worldwide, to discover natural products that can suppress or prevent the process of carcinogenesis [9-12] and [13]. Research on plants have not only confirmed the presence of potential anticancer components in many plants used traditionally for the treatment of cancer, but also helped in the identification of compounds with anticancer activity from non-traditionally used plants, that have subsequently been developed into clinically useful drugs [14] and [15].

A recent short-term study by Iddamaldeniya *et al*. [1], has shown that diethylnitrosamine (DEN)-induced expression of the p-isoform of glutathione S-transferase (GST-P) in rat livers could be significantly inhibited by treatment of rats for 10 weeks, with the decoction (DC) comprised of *N.sativa *seeds, *H.indicus *roots, and *S. glabra *rhizome, that has also been used in the present investigation. From results obtained, it was concluded that the DC has the potential to protect against chemically-induced hepatocarcinogenesis. Results of the present investigation provide further supportive evidence for this view. Thus, long-term treatment (for up to 16 months) of rats with the DC has been demonstrated to inhibit not only DEN-induced GST-P expression, but also the carcinogen-mediated development of overt tumours (OT) and histopathological changes leading to tumour development (HT) as assessed both by visual observations and by microscopic examination of liver sections stained with H&E, Sweet's silver stain and the PAS stain for glycogen. The above stains have been used by many other researchers to assess histopathological changes associated with liver tumour development [16,17] and [18].

One of the most notable features observed in livers of rats treated only with DEN, was the extensive angiogenesis associated with carcinogen-mediated tumours and tumour-like lesions. In rats treated with DEN and the DC, a marked reduction of angiogenesis was observed. Inhibition of angiogenesis is generally considered to be one method by which tumour growth can be inhibited or reversed [19-21] and [14]. Recent investigations have shown that inhibition of angiogenesis is a mechanism by which many phytochemicals also mediate their anti-cancer effects [22-26] and [27]. The anti-angiogenic effects of these natural products have been shown to be related to their abilities to reduce inflammation and/or vascular permeability, or production of detrimental eicosanoids and other angiogenic factors [28]. The mechanism/s by which the DC comprised of *N. sativa, H. indicus *and *S. glabra *inhibits angiogenesis are not clear, although each of the above plants have been reported to possess strong anti-inflammatory properties [29-31] and [32]. Further studies have to be conducted before any definite conclusions can be reached regarding the mechanism/s by which the DC mediates anti-angiogenic effects.

Previous investigations have shown that *N. sativa *seeds and *H. indicus *root contain components with strong antioxidant activity [33-35] and [36]. All three of the plants in the DC have also been shown to possess immunomodulatory properties [37-40] and [33]. These properties may also contribute to the antihepatocarcinogenic actions of the DC.

Recent in-vivo studies have shown that active principles isolated from *N.sativa *seeds and an *H.indicus *root extract can inhibit tumour development in mouse skin [41] and [42]. Principles isolated from *N.sativa *seeds [41,43] and [39] and *S.glabra *rhizome [44] has also been demonstrated to be cytotoxic to several human cancer cell lines. At present, it is not possible to be certain whether only one of the plants in the DC is mainly responsible for mediating the observed anti-hepatocarcinogenic effects or whether all three plants contribute to different extents. Studies currently being conducted in our laboratory with extracts of the individual plants in the DC, would hopefully in the near future, yield results that would help to clarify these doubts.

## Abbreviations

DEN: Diethylnitrosamine

GST-P: Glutathione S-transferase P-isoform

DAB: Diaminobenzidine

ABC: Avidin Biotin-peroxidase Complex

PH: Partial-Hepatectomy

S.E.M: Standard Error of Mean

OT: Overt Tumours

TL: Tumour like Lesions

HT: Histopathological changes leading to development of tumours

DC: Decoction

DW: Distilled Water

NS: Normal Saline

i.p: Intraperitoneally

PAS: Periodic Acid Schiff

## Authors' contributions

SSI did feed preparation, animal handling and feeding under the supervision of MGT. SSI and MGT participated in the surgical procedure for partial hepatectomy. SSI under the supervision of NR performed the immunohistochemical and histopathological staining processes and the interpretation of the stained sections. IT, NW and SSI conceived, designed the study and IT coordinated it. IT and SSI participated in writing the manuscript.
